# Unveiling a hidden threat: whole genome sequencing exposes the cross-hospital spread of extensively drug-resistant *Pseudomonas aeruginosa*

**DOI:** 10.3389/fmicb.2025.1741917

**Published:** 2026-01-13

**Authors:** Edwin Kamau, Brendan Jones, Tatdanai Kitjawat, Han Ha Youn, Michael J. Schweikert, Angela Caldwell, Christopher Harens, Patrick T. Kiernan, John Mark Velasco, Francois Lebreton, Nathanial K. Copeland

**Affiliations:** 1 Tripler Army Medical Center, Honolulu, HI, United States; 2 Multidrug Resistant Organism Repository and Surveillance Network, Bacterial Diseases Branch, CIDR, Walter Reed Army Institute of Research, Silver Spring, MD, United States; 3 Naval Medical Center San Diego, San Diego, CA, United States; 4 Walter Reed Army Institute of Research – Armed Forces Research Institute of Medical Sciences, Quezon City, Philippines; 5 National Institutes of Health, University of the Philippines Manila, Ermita, Manila, Philippines

**Keywords:** healthcare transmission, infection control, multidrug-resistant and extensively drug-resistant, *Pseudomonas aeruginosa*, ST235 clone, whole genome sequencing

## Abstract

**Introduction:**

Whole genome sequencing (WGS) was performed on 10 extensively drug-resistant (XDR) *Pseudomonas aeruginosa* ST235 isolates to investigate potential healthcare-associated transmission of XDR P. aeruginosa ST235 within a U.S. military treatment facility (MTF), assess its environmental persistence, and determine its genomic relatedness to global strains.

**Methods:**

Patient samples were collected and processed using standard clinical protocols. To assess potential environmental contamination, ICU sink drains from rooms previously occupied by study patients were swabbed, cultured in BHI and BAP media, and screened by PCR targeting 16S rRNA and *P. aeruginosa ecfX*. Isolates included 2 from the index patient with prior hospitalization in the Philippines, 4 from a secondary patient in the same ICU, and 4 from the index patient’s room sink collected 9 months later. Genetic relatedness was evaluated using core genome multilocus sequence typing (cgMLST) and single nucleotide polymorphism (SNP) analysis. Antimicrobial resistance genes were identified with AMRFinderPlus, and global phylogenetic comparisons were performed using publicly available genomes from NCBI and the MRSN database.

**Results:**

The 6 clinical isolates differed by only 0–4 SNPs, indicating high genetic relatedness. The 4 environmental isolates differed from the clinical isolates by just 1 allele in cgMLST and 0–4 SNPs. Global phylogenetic analysis showed close clustering with ST235 strains from the Philippines, with the closest match being a 2018 Manila isolate (1–12 allele differences). The isolates carried the blaVIM-2 carbapenemase gene and exhibited resistance to all tested antibiotics.

**Discussion:**

WGS surveillance revealed an undetected intra-hospital transmission of XDR P. aeruginosa ST235 and its persistence in the environment for at least nine months. The findings underscore the importance of genomic surveillance in identifying AMR outbreaks, especially among patients returning from high-risk regions, and proactive infection control measures to prevent the spread of high-risk AMR clones in healthcare settings.

## Introduction

The increasing prevalence of multidrug-resistant (MDR) and extensively drug-resistant (XDR) *Pseudomonas aeruginosa* in healthcare environments remains a significant concern (Del Barrio-Tofiño et al., 2020; Oliver et al., 2015). The globally disseminated high-risk clone sequence type (ST)235, originating from independent European lineages (Treepong et al., 2018), is particularly worrisome due to its virulence, linked in part to the type III secretion system effector exotoxin. It can harbor over 60 different β-lactamase variants, including multiple carbapenemases, and is associated with high mortality rates (Del Barrio-Tofiño et al., 2020; Juan et al., 2017). Furthermore, ST235 exhibits strong biofilm formation (Mulet et al., 2013; Tahmasebi et al., 2022), facilitating colonization of hospital environments, notably plumbing systems, increasing nosocomial transmission risks (Cahill et al., 2023; Weingarten et al., 2018). The robust biofilm formation in *P. aeruginosa* is mediated by quorum-sensing systems (Las/Rhl/Pqs) and exopolysaccharide production, which enable persistence in hospital plumbing and medical devices (Mulet et al., 2013; Tahmasebi et al., 2022). Lipopolysaccharide (LPS) and alginate capsule contribute to immune evasion and chronic infection, while secreted factors such as LasB elastase, LasA protease, alkaline protease, and rhamnolipids promote tissue destruction and impair host defenses (Anderson et al., 2024). Additional virulence mechanisms include type III secretion system effectors, notably ExoU, associated with cytotoxicity and poor clinical outcomes, as well as siderophore-mediated iron acquisition and outer membrane vesicles that enhance competitiveness (Anderson et al., 2024; Curran et al., 2004). Epidemiologically, ST235 has emerged as the most globally disseminated *P. aeruginosa* high-risk clone, reported across Asia, Europe, and the Americas, and frequently linked to outbreaks in intensive care units and invasive device-related infections (Qin et al., 2022; Treepong et al., 2018). Recent surveillance indicates ST235 often carries ExoU and O11 serotype, correlating with severe disease and high mortality (Recio et al., 2020).

Early diagnostic tools for high-risk clones are urgently needed to guide clinical management and infection control (Mulet et al., 2019; Pelegrin et al., 2019; Pincus et al., 2020). Whole genome sequencing (WGS) surveillance is invaluable for detecting outbreaks and healthcare transmissions (Stribling et al., 2024), enhancing patient safety and yielding cost savings (Kracalik et al., 2022; Sundermann et al., 2022; Sundermann et al., 2023), exemplified by the recent XDR *P. aeruginosa* outbreak from contaminated artificial tears (Sundermann et al., 2023).

U.S. Service Members (SMs) are exposed to high AMR rates globally, potentially affecting their operational readiness and contributing to the spread of resistant pathogens upon their return to U.S. healthcare facilities. This report describes WGS surveillance identifying an undetected healthcare transmission of an XDR *P. aeruginosa* ST235 clone from a SM (index patient) to a second patient and the hospital environment after the SM received initial care in the Philippines and subsequent treatment across multiple U.S. facilities.

## Materials and methods

Patient data was collected from electronic medical records. Clinical samples were obtained as part of routine care and processed in CAP-accredited laboratories. Environmental sampling of ICU drainage systems involved vigorous swabbing of sink drain plates, followed by inoculation in enriched blood heart infusion (BHI) broth and on blood agar plates (BAP). BHI samples underwent reverse transcription PCR targeting bacterial 16S rRNA and the *P. aeruginosa ecfX* gene as previously described (Clifford et al., 2012). The *P. aeruginosa ecfX* gene primers are species specific and have demonstrated efficiency of 93.8% with an R2 value of 99.1%. Briefly, primers were designed using Primer Express version 2.0 (Applied Biosystems, Carlsbad, CA) with an optimal annealing temperature of 56°C. Real-time PCR reactions were performed in 20 μL volumes with 200 nM primers. *P. aeruginosa*-positive BHI cultures led to the isolation and sequencing of multiple colonies from blood agar.

Genomic DNA was extracted, and libraries were prepared as previously described (Anderson et al., 2024; Galac et al., 2020; Mills et al., 2022). Briefly, DNA was extracted using the DNeasy UltraClean microbial kit (Qiagen, Germantown, MD, United States), and libraries were constructed using the Kapa HyperPlus library preparation kit (Roche Diagnostics, Indianapolis, IN, United States). Libraries were quantified using the Kapa library quantification kit Illumina/Bio-Rad iCycler (Roche Diagnostics) on a CFX96 real-time cycler (Bio-Rad, Hercules, CA, United States). For the MiSeq, libraries were normalized to 2 nM, pooled, denatured, and diluted to 20 pM. The pooled samples were further diluted to a final concentration of 14 pM. Samples were sequenced using MiSeq reagent kit v3 (600 cycles; 2 × 300 bp) (Illumina). For the NextSeq, libraries were normalized to 1 nM, pooled, denatured, and diluted to 20 pM. The pooled samples were further diluted to a final concentration of 2.1 pM. Samples were sequenced using NextSeq reagent kit 500/550 v2 (300 cycles; 2 × 150 bp) (Illumina). Raw reads were processed using bbduk for adapter removal and quality trimming, and Kraken2 v2.1.2 for initial taxonomic assignment and contamination screening. *De novo* draft genome assemblies were generated using shovill, with coverage estimated by bbmap (minimum contig size 200 bp, coverage ≥ 49.5). *In silico* MLST was performed using the scheme developed by Curran et al. (2004), and gene annotation was performed with Bakta, and antimicrobial resistance genes were predicted using AMRFinderPlus. Sequence type (ST) was determined using mlst.

Genetic relatedness was assessed using core genome MLST (cgMLST) in SeqSphere + Software (Curran scheme, 90% cutoff). For finer-scale analysis within cgMLST clusters, SNP analysis was conducted using Snippy and Pilon (error correction), followed by Gubbins to filter out recombination, using the earliest temporal isolate as a reference. Publicly available *P. aeruginosa* ST235 WGS data from the NCBI database was included for comparative analysis. Phylogenetic tree construction, SNP matrix generation, average nucleotide identity (ANI) calculation, and accessory genome profiling were performed using CLC Genomic Workbench (Qiagen, Valencia, CA, United States). All ST235 genomes sequenced in this study have been deposited in the NCBI under BioProject PRJNA1259075^1^.

## Results

**FIGURE 1:**
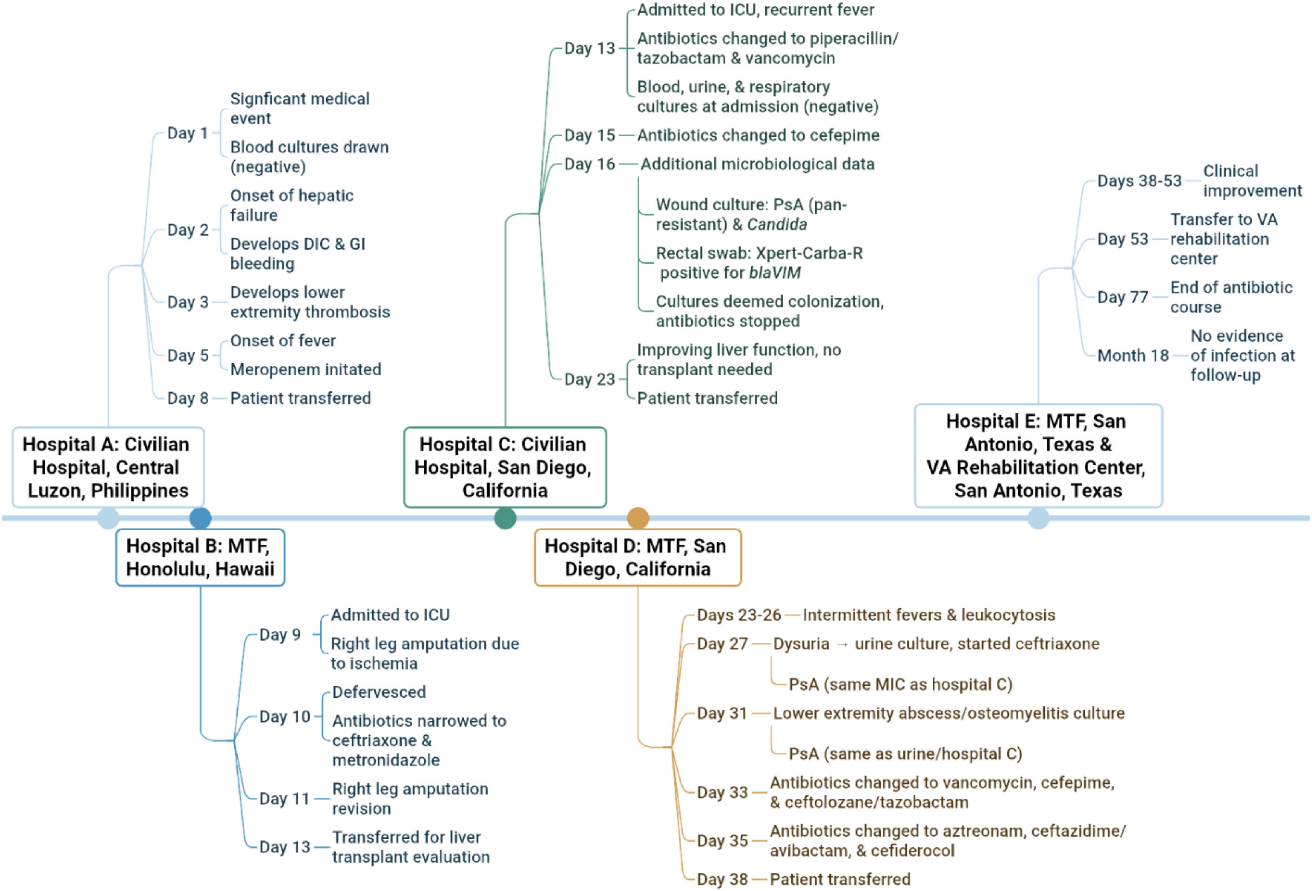
A summary of the index patient’s treatment course. After emergent evacuation to a hospital in the Philippines, the patient was transferred to 4 other hospitals and a rehabilitation center, covering over 9,000 miles.: DIC, disseminated intravascular coagulation; GI, gastrointestinal; ICU, intensive care unit; MIC, minimum inhibitory concentration; MTF, military treatment facility; PsA, *Pseudomonas aeruginosa*; VA, Veterans Affairs.

The index patient, a 21-year-old active-duty female Sailor with no significant past medical history, was emergently evacuated from the Philippines due to toxic medication ingestion, presenting with shock, severe acidosis, acute renal failure requiring dialysis, fulminant hepatic failure, disseminated intravascular coagulation, and significant gastrointestinal bleeding. Initially treated with meropenem, she developed lower extremity thrombosis necessitating below-knee amputation (Figure 1). Initial blood and urine cultures were negative upon arrival at a U.S. military treatment facility (MTF), a large tertiary US military hospital located outside the continental United States. On illness day 16, a wound culture yielded XDR *P. aeruginosa*, demonstrating non-susceptibility to all tested antibiotics (detailed MICs in Table 1), and *Candida parapsilosis*. A rectal swab was positive for the *bla*_*VIM*_ gene, indicating VIM carbapenemase production. Following clinical improvement, she was transferred to another MTF, a large tertiary US military hospital located in the continental United States.

**TABLE 1:** Minimum inhibitory concentration testing results of different antibiotics that were tested against the *Pseudomonas aeruginosa* isolated from a wound culture obtained on illness day 16.

Antibiotics	MIC (μ g/mL)	MIC interpretation	DD Zone (mm)	DD interpretation
Amikacin	≥ 64	R		
Cefazolin	≥ 64	R		
Cefepime	≥ 64	R		
Cefiderocol			16 mm	I
Ceftazidime	≥ 64	R		
Ciprofloxacin	≥ 4	R		
Gentamicin	≥ 16	R		
Levofloxacin	≥ 8	R		
Meropenem	≥ 16	R		
Tobramycin	≥ 16	R		
Ceftazidime/avibactam			8 mm	R
Ceftolozane/tazobactam			6 mm	R

DD, disk diffusion; I, intermediate; MIC, minimal inhibitory concentration; R, resistant.

Subsequently, the index patient developed a urinary tract infection on illness day 27, with urine cultures growing *Citrobacter youngae*, *Klebsiella pneumoniae*, and XDR *P. aeruginosa* exhibiting an identical resistance profile to the initial isolate (Table 1) and confirmed to be *bla*_*VIM*_ positive. Osteomyelitis was suspected at the amputation site, and operative cultures from incision and drainage procedures also grew XDR *P. aeruginosa* with the same resistance pattern. She received various broad-spectrum antibiotics and underwent revision above-knee amputation. After further treatment, she was transferred to a third MTF, also a large tertiary US military hospital located in the continental United States and eventually completed a 6-week antibiotic course with no further infection issues during 18 + months of follow-up (Figure 1).

The secondary patient, a 48-year-old male military dependent with a history of anoxic brain injury and recurrent UTIs, was admitted for *K. pneumoniae* septic shock 1 day prior to the index patient being placed in adjacent ICU rooms in the first MTF. His hospitalization was complicated by multiple infections, and after several weeks, both urine and respiratory cultures grew XDR *P. aeruginosa*. Despite intensive care, he developed multiorgan failure and died on hospital day 40.

**FIGURE 2:**
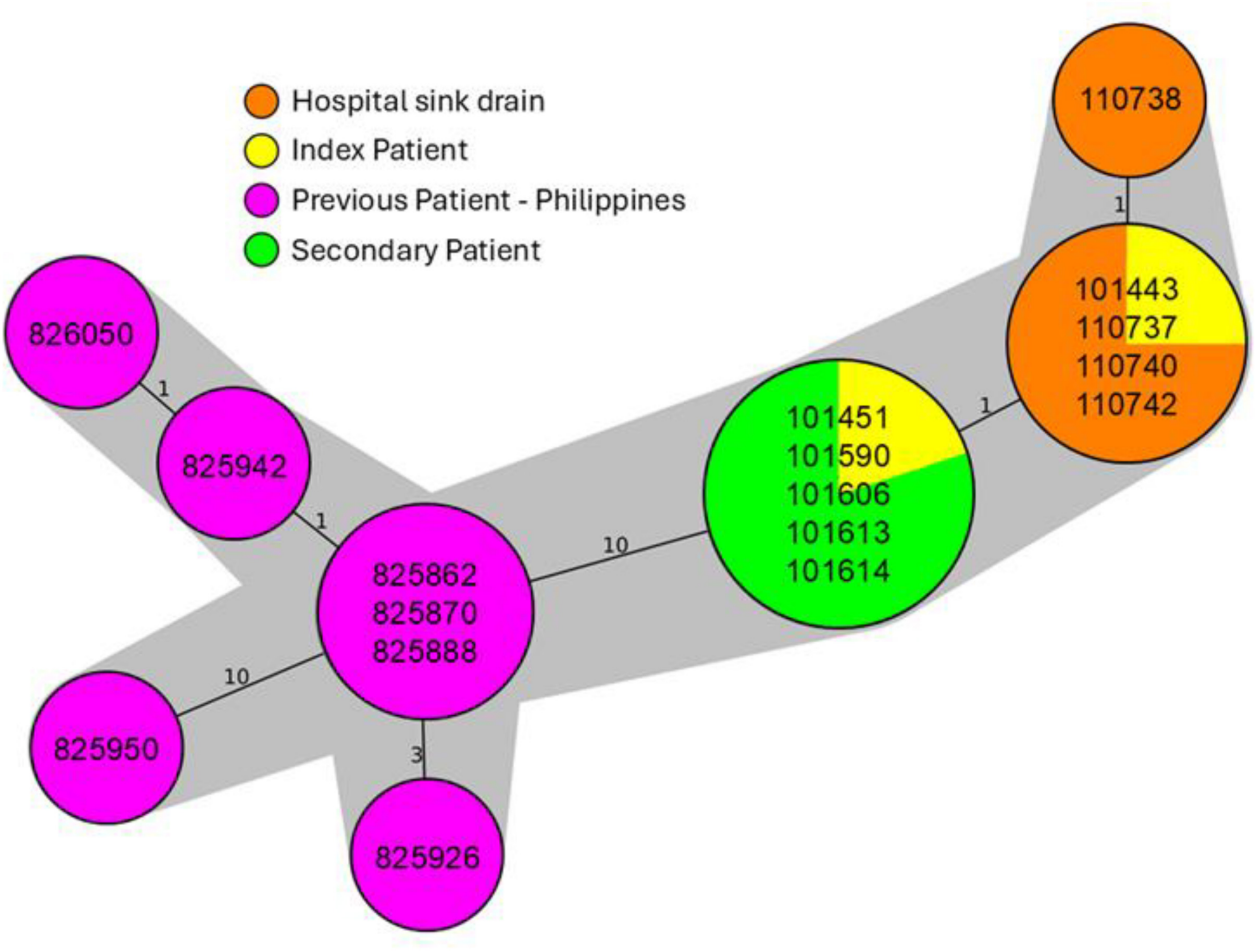
Diagram of the genetic relatedness between patient and environmental *P. aeruginosa* isolates from our study. Minimum spanning tree generated using cgMLST allelic profiles. The numbers on each line indicate the number of allelic differences between each isolate. Each study isolate is shown inside the circles in unique MRSN #. Isolates with no allelic differences occupy the same circle; isolates separated by 1–10 allelic differences (i.e., highly related) are highlighted with gray shading.

**FIGURE 3:**
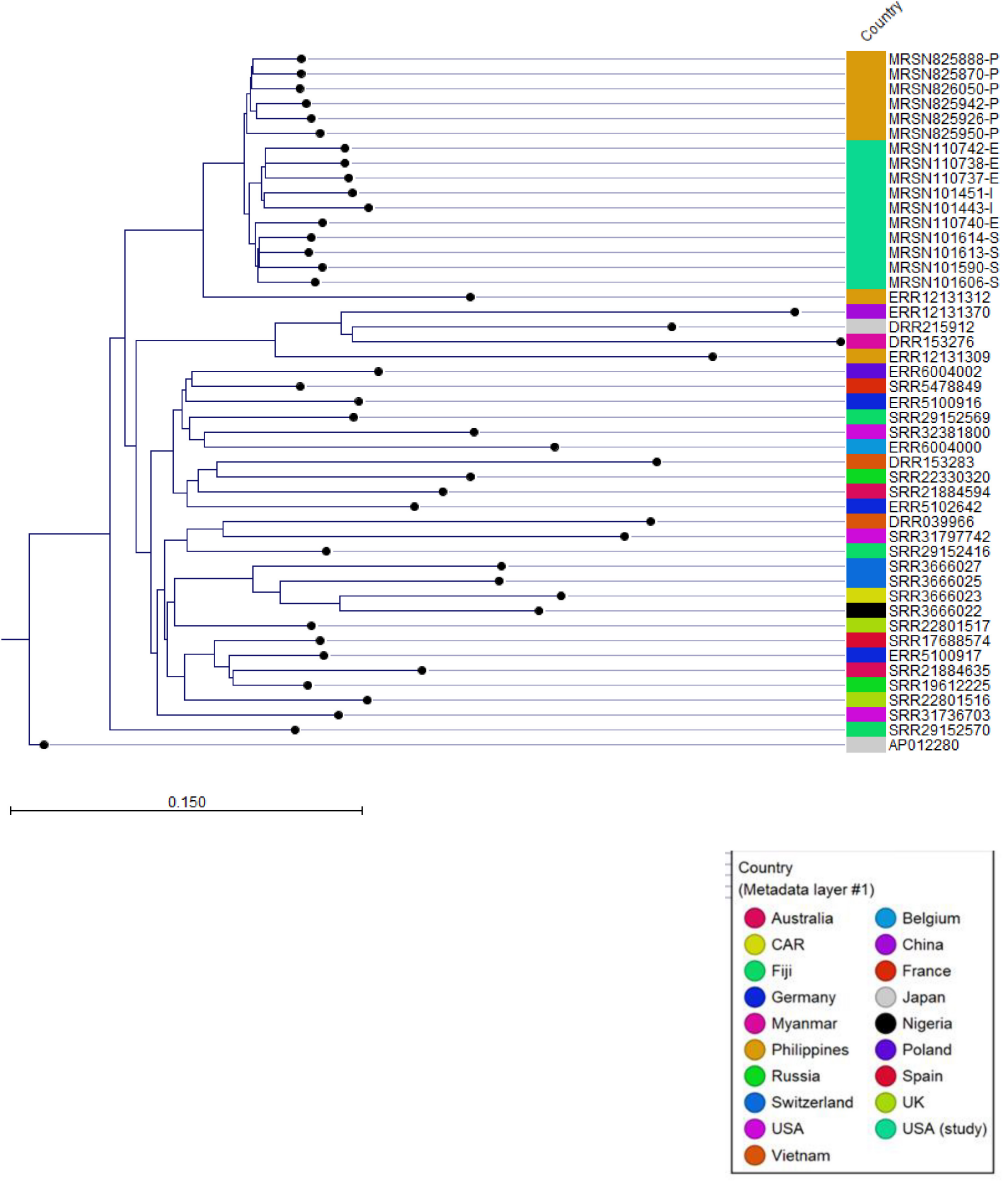
Global Phylogenetic Analysis of *Pseudomonas aeruginosa* ST235 Isolates. A phylogram-based Neighbor-Joining tree illustrating the genetic relationships among *P. aeruginosa* ST235 isolates, including those from this study. *K-mers* (length 16) were extracted using the *K-mer* Based Tree Construction tool in CLC Genomic Workbench, and pairwise distances were calculated using the Jaccard Distance method. The resulting distance matrix was used to construct the phylogenetic tree, where branch lengths reflect genetic divergence. Distinct clusters indicate evolutionary relatedness, with our isolates clustering closely with samples from the Philippines and other Asian countries. This suggests potential regional transmission dynamics and persistence of genetically similar strains in healthcare environments. Study samples are from the index case (- I), secondary case (- S), or the environment (- E) denoted at the end of the sequence name. CAR, Central African Republic; UK, United Kingdom; USA, United States of America.

Comparative genomic analysis of the two clinical *P. aeruginosa* isolates from the index patient and the four from the secondary patient identified them as ST235 and revealed high genetic relatedness, with only 0–4 single nucleotide polymorphisms (SNPs) differentiating them (Figure 2 and Supplementary Figure). These isolates all carried several antimicrobial resistance genes, including the *bla_*VIM*–2_* carbapenemase gene, consistent with the observed phenotypic resistance (Table 2). Environmental sampling of the index patient’s ICU room sink, conducted 9 months after her treatment, yielded four *P. aeruginosa* ST235 isolates that were also highly genetically related to the patient isolates, differing by only a single allele in cgMLST and 0–4 SNPs (Figure 2; Table 3 and Supplementary Figure). Global phylogenetic analysis demonstrated that the study isolates clustered closely with other ST235 genomes from the Philippines and other Asian countries (Figure 3), with the closest matches in the MRSN database being ST235 strains from the Philippines collected in 2018 (1–12 allele differences). The isolates from this study differed from those 2018 Philippine MRSN isolates by 21–32 SNPs (Supplementary Table).

**TABLE 2:** Antibiotic resistance genes carried by all ST-235 *P. aeruginosa.*

Gene^1^	Predicted phenotype^2^
*aac(6′)-Ib3*	Aminoglycosides: Amikacin, Kanamycin, Tobramycin
*ant(2″)-Ia*	Aminoglycosides: Gentamicin, Kanamycin, Tobramycin
*aph(3′)-IIb*	Aminoglycosides: Kanamycin
*aadA1*	Aminoglycosides: Streptomycin
*blaVIM-2*	β-lactams: Carbapenems
*blaPDC-35*	β-lactams: Cephalosporins
*blaOXA-10*	β-lactams: Penicillins, Early Cephalosporins
*blaOXA-488*	β-lactams: Penicillins, Early Cephalosporins
*crpP*	Fluoroquinolones
*fosA*	Fosfomycin
*catB3*	Phenicols
*catB7*	Phenicols
*cmlA1*	Phenicols
*sul1*	Sulfonamide

^1^Best hit gene based on sequence identity and coverage.

^2^Predicted resistance pattern based on antibiotic resistance gene product.

**TABLE 3:** A single nucleotide polymorphism (SNP) matrix showing SNP difference between the isolates from the index and secondary patients, and environmental.

Sample name	Sample number	1	2	3	4	5	6	7	8	9	10
MRSN101443-I	1	0	1	3	2	4	2	0	0	1	0
MRSN101451-I	2	1	0	2	1	3	1	1	1	2	1
MRSN101590-S	3	3	2	0	1	1	1	3	3	2	3
MRSN101606-S	4	2	1	1	0	2	0	2	2	1	2
MRSN101613-S	5	4	3	1	2	0	2	4	4	3	4
MRSN101614-S	6	2	1	1	0	2	0	2	2	1	2
MRSN110737-E	7	0	1	3	2	4	2	0	0	1	0
MRSN110738-E	8	0	1	3	2	4	2	0	0	1	0
MRSN110740-E	9	1	2	2	1	3	1	1	1	0	1
MRSN110742-E	10	0	1	3	2	4	2	0	0	1	0

There were 0–4 SNP difference between the isolates. Whole genome sequencing data in FASTQ format were uploaded into CLC Genomics Workbench (Qiagen) along with the reference genome AP012280. Reads were mapped to the reference genome using default settings. Variants were identified with the Fixed Ploidy Variant Detection tool, using a minimum coverage of 10x and a variant frequency threshold of 20%. The resulting variants were used to generate a SNP matrix with the Create SNP Tree tool, displaying pairwise SNP differences between samples. A SNP matrix showing SNP difference between the different clinical and environmental ST235 isolates. There were 0–4 SNP difference between all the isolates. I—index patient; S—secondary patient; E—environment.

## Discussion

This study highlights the critical utility of a genomic sequence-based surveillance program in identifying a previously unrecognized outbreak of an XDR *P. aeruginosa* ST235 clone, demonstrating both intra- and interhospital transmission. The genomic evidence strongly suggests that the outbreak likely originated with the index patient’s exposure in the Philippines before spreading to the first MTF, subsequently transmitting to a second patient in close proximity, and importantly, persisting in the healthcare environment for at least 9 months within the index patient’s room sink. These findings underscore the significant challenges associated with the detection and management of infections or colonizations caused by MDR and XDR organisms, particularly high-risk clones like ST235. The study emphasizes the considerable risk of transmission posed by this clone to other vulnerable patients and highlights its capacity for robust biofilm formation, which likely contributed to its prolonged persistence within the hospital plumbing system.

Crucially, this investigation reinforces the necessity for heightened vigilance regarding the spread of antimicrobial-resistant pathogens. This includes recognizing the clinical significance of patient colonization with MDR/XDR organisms, as these can serve as silent reservoirs for transmission within healthcare settings. For patients being transferred or returning from regions with high rates of AMR – especially those with a history of hospitalization or invasive procedures – enhanced surveillance protocols, rigorous contact isolation measures, and, where clinically appropriate, decolonization strategies should be prioritized, even in the absence of initial positive culture results. This proactive approach is essential to effectively curb the dissemination of AMR pathogens and safeguard patient safety in healthcare environments.

The successful detection of this outbreak was directly enabled by the implementation of a genomic sequence-based surveillance program that spanned multiple healthcare facilities across continents. This approach not only facilitated the identification of the transmission events but also provided critical information that supported the targeted removal of ST235-containing biofilm from the hospital environment. Furthermore, it informed the adoption of specific infection prevention and control practices aimed at mitigating future transmissions and enhancing overall infection control measures within the facility. The established value of WGS-based surveillance in detecting outbreaks and providing clinically actionable insights is further supported by our findings, which directly link the transmission of the *P. aeruginosa* ST235 clone, including its introduction into our hospital, its subsequent spread to another patient, and its prolonged survival within the hospital environment. Notably, the discovery, tracking, and ultimate eradication efforts targeting this clone – including its presence in sink drain biofilms – were only feasible through the application of WGS-based sequencing technologies.

Comparison of the study isolates with over 3,500 *P. aeruginosa* genomes within the MRSN database revealed the closest genetic relationship to a blaVIM-2-positive *P. aeruginosa* ST235 strain isolated in 2018 from the urine of a female patient hospitalized in Manila, Philippines. Similarly, comparative analysis with the global NCBI database corroborated this finding, demonstrating that our isolates clustered closely with other ST235 genomes originating from the Philippines, as well as other countries in Asia (Figure 3).

The ST235 sublineage, which emerged in Europe in the mid-1980s, has since become the most geographically widespread and concerning high-risk *P. aeruginosa* clone globally. It is characterized by its extensive antimicrobial resistance profile and its pronounced ability to form robust biofilms (Del Barrio-Tofiño et al., 2020; Oliver et al., 2015; Treepong et al., 2018). These combined traits enable ST235 and similar high-risk clones to readily establish themselves within healthcare environments, underscoring the importance of proactive surveillance of incoming patients from high-risk regions and routine environmental monitoring. The development and implementation of molecular diagnostic tools specifically targeting high-risk *P. aeruginosa* clones would significantly enhance these surveillance efforts.

In addition to robust biofilm formation, high-risk ST235 clones commonly harbor type III secretion system (T3SS) effectors, especially ExoU, which is strongly linked to cytotoxicity and poor clinical outcomes; ST235’s pathogenicity has repeatedly been associated with an exoU-positive genotype and O11 serotype. Quorum-sensing (QS) networks (Las/Rhl/Pqs) coordinate production of exoproteases (LasB elastase, LasA), alkaline protease (AprA), phenazines (e.g., pyocyanin), and rhamnolipids, all contributing to tissue damage, immune evasion, and biofilm maturation; recent work highlights rhamnolipid micelles as highly cytotoxic to host cells and correlated with virulence across clinical isolates (Qin et al., 2022). ST235 also shows accessory genome diversity (e.g., GI1/GI2 islands) and variable QS phenotypes; comparative analyses indicate some ST235 representatives display high constitutive T3SS activity with attenuated QS signaling, further emphasizing clonal heterogeneity in virulence strategy (Qin et al., 2022). Finally, type VI secretion (T6SS), siderophores (pyoverdine/pyochelin), and outer membrane vesicles contribute to ST235 competitiveness and host damage, underscoring the multi-factorial nature of virulence in this lineage (Qin et al., 2022; Recio et al., 2020).

The presence of ST235 in healthcare environments presents three major interconnected challenges. First, the extensive drug resistance exhibited by ST235 significantly complicates treatment, often limiting therapeutic options to last-resort antibiotics, which are associated with increased adverse effects and higher healthcare costs (Loconsole et al., 2020). Second, effective infection control is considerably hampered by the ability of ST235 to form persistent biofilms within healthcare settings, thereby elevating the risk of nosocomial infections. This necessitates the implementation of stringent and comprehensive infection control strategies, including enhanced environmental cleaning and disinfection protocols, coupled with thorough environmental monitoring (Sendra et al., 2024). Finally, the inherent outbreak potential of ST235 poses a substantial threat to patient safety within healthcare facilities due to its combination of high virulence and extensive resistance. Rapid and accurate detection, followed by prompt and effective intervention measures, are therefore essential to prevent widespread transmission (Gray et al., 2023).

The identification of this transmission cluster prompted the immediate implementation of routine screening and isolation policies at our facility to proactively mitigate the further spread of antimicrobial-resistant pathogens colonizing patients. These enhanced policies now include peri-rectal surveillance cultures and empiric contact precautions for all patients being transferred from areas with potentially elevated rates of antimicrobial resistance. Our facility serves as the primary Military Health System referral center for the entire Indo-Pacific region, encompassing over 400,000 beneficiaries. The geographical proximity of Hawaii to regions with high AMR prevalence, coupled with its status as a major international tourist destination, further amplifies its inherent risk for the introduction and subsequent spread of AMR pathogens. As clearly highlighted by the findings of this study, Hawaii serves as a significant gateway to the continental U.S., underscoring the critical and ongoing need for vigilant surveillance efforts and collaborative partnerships with civilian healthcare facilities and the state Department of Health to effectively prevent the introduction and dissemination of antimicrobial-resistant pathogens within our communities.

In this study, we did not perform PAO1/PA14 clade assignment. Notably, phylogenomic studies place ST235 within *P. aeruginosa* sensu stricto and show that ST235 comprises multiple subclades (C1–C14) with geographic structure and is genetically distinct from PA14 (ST253) despite frequent shared exoU carriage (Fischer et al., 2020).

## Conclusion

WGS surveillance proved critical for detecting an unrecognized outbreak of XDR *P. aeruginosa* ST235, tracing its likely origin to the Philippines and subsequent spread across facilities and the environment. These findings underscore the need for early genomic surveillance, strict infection control, and proactive measures for patients from high-AMR regions. Widespread adoption of WGS-based programs is essential to prevent transmission of high-risk clones and protect patient safety.

## Data Availability

The original contributions presented in the study are publicly available. This data can be found here: https://www.ncbi.nlm.nih.gov/bioproject/1259075.

## Footnotes

1
https://www.ncbi.nlm.nih.gov/bioproject/1259075

## Author disclaimer

The views expressed in this study are those of the authors and do not necessarily reflect the official policy or position of the Defense Health Agency, Department of Defense, nor the U.S. Government. This work was prepared as part of official duties. Title 17, U.S.C., Section 105 provides that copyright protection under this title is not available for any work of the U.S. Government. Title 17, U.S.C., Section 101 defines a U.S. Government work as a work prepared by a military service member or employee of the U.S. Government as part of that person’s official duties.
