# Supplementary material for: Unveiling a hidden threat: whole genome sequencing exposes the cross-hospital spread of extensively drug-resistant Pseudomonas aeruginosa
Source: Front Microbiol. 2026 Jan 13;16:1741917. doi: 10.3389/fmicb.2025.1741917 (PMC12835893; doi:10.3389/fmicb.2025.1741917)
Supplement: Supplementary file 1 [file Data_Sheet_1.docx]

**Title:** Unveiling a Hidden Threat: Whole Genome Sequencing Exposes the Cross-Hospital Spread of Extensively Drug-Resistant *Pseudomonas aeruginosa*

**Authors:** ^1^Edwin Kamau, ^2^Brendan Jones, ^1^Tatdanai Kitjawat, ^1^Han Ha Youn, ^1^Michael J. Schweikert, ^1^Angela Caldwell, ^1^Christopher Harens, ^3^Patrick T. Kiernan, ^4,5^John Mark Velasco, ^2^Francois Lebreton, ^1^Nathanial K. Copeland

^1^Tripler Army Medical Center, Honolulu, HI, USA

^2^ Multidrug resistant organism Repository and Surveillance Network (MRSN), Bacterial Diseases Branch, CIDR, Walter Reed Army Institute of Research (WRAIR), Silver Spring, MD, USA

^3^Naval Medical Center San Diego, San Diego, CA, USA

^4^Walter Reed Army Institute of Research – Armed Forces Research Institute of Medical Sciences (WRAIR-AFRIMS) in Quezon City, Philippines.

^5^National Institutes of Health, University of the Philippines Manila, Ermita, Manila, Philippines.

Corresponding author: Edwin Kamau

Corresponding author email: [edwin.kamau@cepheid.com](mailto:edwin.kamau@cepheid.com)

Key words: Multidrug-resistant and extensively drug-resistant; *Pseudomonas aeruginosa*; Whole genome sequencing; Infection control; ST235 clone; Healthcare transmission.

Short Title: WGS Surveillance of XDR *P. aeruginosa* ST235

Author biography: Experienced Clinical Microbiologist and Medical Director with 20 years of expertise in infectious diseases, clinical microbiology, and strategic leadership. Has special interest in drug resistance and infection prevention and control activities.

**Supplementary Figure**


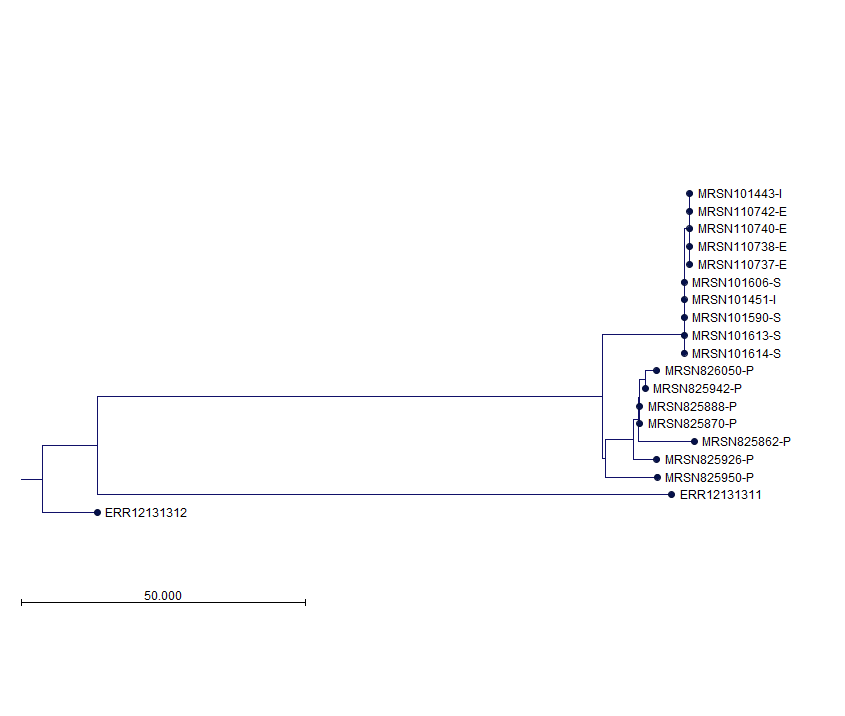


Supplementary Figure. Phylogram-Based Phylogenetic Analysis of *Pseudomonas aeruginosa* ST235 Isolates A phylogram depicting the genetic relationships among *Pseudomonas aeruginosa* ST235 isolates, including those from our study and isolates from the Philippines. Phylogenetic analysis was performed using SNP-based comparisons in CLC Genomic Workbench. Each branch represents a distinct isolate, with branch lengths proportional to the number of SNP differences. Longer branches indicate greater genomic divergence, while shorter branches suggest closer genetic relatedness. The clustering pattern highlights potential transmission dynamics and evolutionary trajectories of the ST235 clone within clinical and regional contexts. The isolates with MRSN # are from our study, including those obtained from a patient in the Philippines in 2018. The other two sequences are from NCBI.

**Supplementary Table**

SNP Matrix Comparing Genetic Relatedness of Study Isolates and Reference Databases


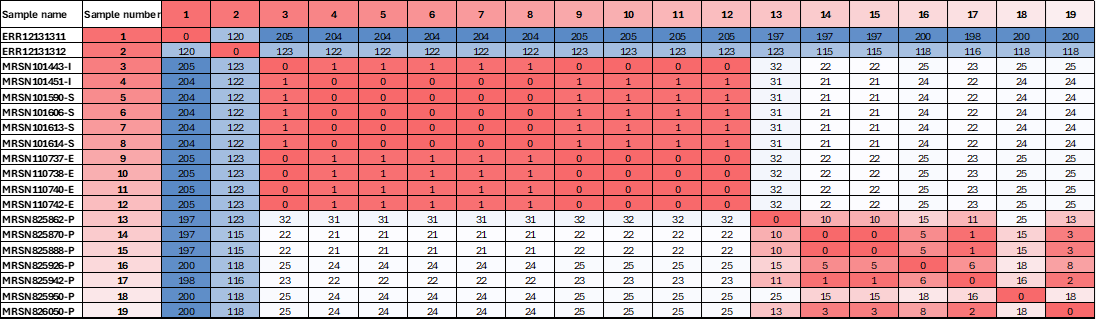


A SNP matrix showing SNP difference between isolates from our study, patient isolates obtained by MRSN from the Philippines in 2018, and two isolates from the Philippines obtained from NCBI.
